# Modifiable “Predictors” of Zinc Status in Toddlers

**DOI:** 10.3390/nu10030306

**Published:** 2018-03-05

**Authors:** Lisa Daniels, Sheila M. Williams, Rosalind S. Gibson, Rachael W. Taylor, Samir Samman, Anne-Louise M. Heath

**Affiliations:** 1 Department of Human Nutrition, University of Otago, Dunedin 9054, New Zealand; lisa.daniels@otago.ac.nz (L.D.); rosalind.gibson@otago.ac.nz (R.S.G.); samir.samman@sydney.edu.au (S.S.); 2 Department of Medicine, University of Otago, Dunedin 9054, New Zealand; rachael.taylor@otago.ac.nz; 3 Department of Preventive and Social Medicine, University of Otago, Dunedin 9054, New Zealand; sheila.williams@otago.ac.nz; 4 School of Life and Environmental Sciences, University of Sydney, Sydney, NSW 2006, Australia

**Keywords:** zinc status, plasma zinc, toddlers, food fussiness, red meat, infant formula, zinc intake, complementary feeding

## Abstract

Suboptimal zinc status is common in very young children and likely associated with increased risk of infection and detrimental effects on growth. No studies have determined potentially modifiable “predictors” of zinc status in toddlers from high-income countries. This cross-sectional analysis of 115 toddlers from the Baby-Led Introduction to SolidS (BLISS) study used weighed diet records (three non-consecutive days) to assess dietary intake, and a venous blood sample (trace-element free techniques) to assess plasma zinc, at 12 months of age. “Predictors” of plasma zinc were determined by univariate analysis and multiple regression. Mean (SD) plasma zinc was 9.7 (1.5) μmol/L, 60% were below the IZiNCG reference limit of <9.9 μmol/L. Median (25th, 75th percentiles) intake of zinc was 4.4 (3.7, 5.4) mg/day. Red meat intake (*p* = 0.004), consumption of zinc-fortified infant formula (3–6 mg zinc/100 g) (*p* = 0.026), and food fussiness (*p* = 0.028) were statistically significant “predictors” of plasma zinc at 12 months. Although higher intakes of red meat, and consumption of infant formula, are potentially achievable, it is important to consider possible barriers, particularly impact on breastfeeding, cost, and the challenges of behavior modification. Of interest is the association with food fussiness—further research should investigate the direction of this association.

## 1. Introduction

During early childhood the risk of zinc deficiency is increased [1], primarily because of a higher physiological requirement for zinc due to the high growth rate during this time [2]. However, zinc intake is also an issue because the complementary foods typically offered when solids are introduced (e.g., fruit, vegetables, cereals) are generally low in absorbable zinc, and because after six months of age breast milk no longer provides sufficient zinc to meet requirements [3].

Suboptimal zinc status has frequently been reported in studies of young children from high-income countries, including New Zealand [4,5,6,7,8,9,10,11,12,13]. Inadequate zinc status during early childhood is associated with an increased risk of infection [14,15]. This is particularly important as many toddlers participate in child care programs where the exposure to illness is high [16]. Inadequate zinc status can also have detrimental effects on growth [17]. Therefore, it is also important to determine what factors may be modifiable during early childhood that might improve the zinc status of this age group.

Several international studies have indicated that various biological and methodological factors affect zinc status, including age [5,18,19], season [20,21], inflammation or infection [18,22], time of the day [22,23,24,25], and fasting status [22,24,25]. Yet, to our knowledge, no study has assessed modifiable “predictors” of zinc status in healthy infants or toddlers after standardizing these factors that are known to affect plasma zinc concentrations.

The aim of this paper was to examine associations between dietary, biochemical, and other variables, and plasma zinc concentration, and to determine potentially modifiable “predictors” of plasma zinc at 12 months of age.

## 2. Materials and Methods

### 2.1. Study Design

This is a cross-sectional analysis of data collected in the Baby-Led Introduction to SolidS (BLISS) study [26]. BLISS was a randomized controlled trial investigating the impact of a modified version of Baby-Led Weaning (BLW) on several infant outcomes including growth [27], iron [28], and zinc [29] status, and choking [30]. Written consent was obtained from all adult participants before randomization to one of two groups: BLISS (infant self-feeding using a modified version of BLW) or Control (usual care) between November 2012 and March 2014. The Lower South Regional Ethics Committee of New Zealand approved the study (LRS/11/09/037).

### 2.2. Outcome Assessment

#### 2.2.1. Questionnaire Data

Demographic data, including maternal age, ethnicity, education, and parity, were collected at baseline (late pregnancy) by questionnaire. The participant’s current address was used to determine household deprivation using the New Zealand Index of Deprivation (NZDep) score [31]. Infant sex, birth weight, and gestational age at birth were accessed through hospital records. Parents completed a questionnaire (self-administered) at 12 months of age which included questions from the Children’s Eating Behaviour Questionnaire on food fussiness [32]. Using response options of “never”, “rarely”, “sometimes”, “often”, or “always”, parents indicated whether their child enjoyed tasting new foods, consumed a wide variety of foods, were interested in tasting new foods, refused new foods, decided whether a food was disliked before tasting, and whether there was difficulty in pleasing the toddler with meals. Higher mean scores represent higher levels of food fussiness [32] and Cronbach α for our sample ranged from 0.64 to 0.88 for individual questions.

#### 2.2.2. Anthropometric Assessment

Research staff measured toddler weight (Seca, Model 334, Hamburg, Germany) and length (Rollameter 100c length board, Harlow Healthcare, South Shields, UK) in duplicate when they were 12 months of age. Infants were weighed without clothes (wearing only a standard weight nappy which was subtracted from their weight on data entering), and length was measured with no shoes, complying with the World Health Organization (WHO) protocols [33]. Weight-for-age and length-for-age z-scores were calculated using the WHO child growth standards reference data [34].

#### 2.2.3. Dietary Assessment

Weighed three-day diet records (WDRs) were used to assess dietary intake at 12 months of age. Parent participants were given detailed written and oral instructions for completing the WDR and then recorded all foods and beverages consumed on three randomly assigned non-consecutive days (two weekdays and one weekend day) over a three-week period. Each day of the week was represented approximately an equal number of times among participants to control for day-of-the-week effects. Dietary scales (Salter Electronic, Salter Housewares Ltd., Tonbridge, UK), accurate to ±1 g were given to each participant to complete the WDR. The completed WDRs were entered into Kai-culator (Version 1.13s, University of Otago, Dunedin, New Zealand), a New Zealand dietary analysis software program that includes dietary data from the New Zealand Food Composition Database (FOODfiles 2010, Plant and Food Research) [35]. “Meat, fish, poultry”, heme iron, and non-heme iron [36] and phytate were not available in the New Zealand Food Composition Database, but were determined using values from the literature and information from the manufacturers [29].

#### 2.2.4. Biochemical Assessment

A venous (antecubital vein) blood sample was collected from participants at 12 months of age using trace-element free lithium heparin anticoagulated tubes (7.5 mL; S-Monovette, Sarstedt, Nümbrecht, Germany) and standardized procedures, as recommended by the International Zinc Nutrition Consultative Group (IZiNCG) [37]. A local anesthetic (Ametop gel, Smith & Nephew, London, UK) was applied to the toddler’s arm 1–2 h before the appointment, and infants were seated on a parent’s knee for the phlebotomy. Strict procedures were used to control for known predictors of plasma zinc concentrations: time of blood collection [22,23,24,25], fasting status [22,24,25], and inflammation [18,22]. To standardize food intake prior to the blood test, parents were asked to feed their baby milk (as much as they wanted of the milk they usually have, e.g., breast milk, infant formula, or cow’s milk) 90 min prior to the blood test appointment, and then to give no other food or drink until after the blood test. To reduce the impact of inflammation, if the child was unwell on the day before the scheduled blood test (i.e., presence of fever, diarrhea, or vomiting), the blood test was delayed for 14 days. A questionnaire was also administered at the blood test appointment which asked parents to confirm the timing of the milk feed, whether any illness was present and whether any zinc-containing preparations were used in the past month.

Plasma zinc was determined using flame atomic absorption spectrophotometry (Perkin Elmer AAnalyst 800), in the Department of Human Nutrition, University of Otago. The accuracy and precision of the analyses were checked using certified controls and in-house pooled samples (after every 15 samples). The analysed mean (SD, CV) value for the zinc control (UTAK Laboratories, Inc., Valencia, CA, USA) was 65.8 μmol/L (1.9 μmol/L, 2.9%), compared to the manufacturers’ concentration of 65 μmol/L. Since the samples were collected from the toddlers in the morning, low plasma zinc concentrations were defined as a concentration < 9.9 μmol/L [37]. Plasma zinc concentration is used to indicate “zinc status” in this paper as has been recommended by IZiNCG [37], in the absence of a more appropriate biomarker for determining “zinc status”.

C-reactive protein (CRP; a measure of acute inflammation), and α1-acid glycoprotein (AGP; a measure of chronic inflammation) were analysed using a Cobas C311 automatic electronic analyser (Roche Diagnostics, GmbH, Mannheim, Germany). These analyses were carried out in the Department of Human Nutrition laboratories (University of Otago, Dunedin, New Zealand). The mean (SD, CV) for the CRP control (Roche Diagnostics, GmbH, Mannheim, Germany) was 9.5 mg/L (0.4 mg/L, 4.6%), compared with the manufacturer’s concentration of 9.1 mg/L. The multilevel controls for AGP (Roche Diagnostics, GmbH, Mannheim, Germany) were 0.5 g/L (0.01 g/L, 1.1%) and 0.8 g/L (0.01 g/L, 1.4%), compared with the manufacturer’s concentrations of 0.7 g/L and 1.2 g/L, respectively.

Complete blood count, which includes hemoglobin (Sysmex XE 5000 automatic electronic analyser, Kobe, Japan) was determined by Southern Community Laboratories Ltd. (Dunedin, New Zealand), the local clinical laboratory, where external quality control measures are completed regularly.

### 2.3. Statistical Analysis

There were no statistically significant differences in zinc status between the two study groups (Control vs. BLISS) of the BLISS study [29], so the data were combined to enable this cross-sectional analysis. Medians and lower and upper percentiles (25th and 75th) were used to describe the dietary variables. Participant characteristics are presented as means and standard deviations.

Plasma zinc was adjusted for inflammation using the Biomarkers Reflecting Inflammation and Nutrition Determinants of Anemia (BRINDA) multiple linear regression approach described by Larson and colleagues [38]. The adjustment has two components: slopes of the associations between the inflammation markers and plasma zinc concentration, and difference between observed values and the 10th percentile (to avoid over-adjustment amongst individuals with low levels of inflammation). For the first component, two linear regression models were conducted using the natural log of plasma zinc concentration as the dependent variable and natural logs of CRP and AGP as the independent variables to determine the slope of the association (i.e., β1 and β2). For the second component, the 10th percentile for the natural logs of CRP and AGP were determined and the difference between the natural log of the observed value and this 10th percentile was calculated for each participant (e.g., lnCRPdiff). The adjusted plasma zinc was then calculated for each participant: Adjusted plasma zinc concentration = exp[unadjusted lnplasmazinc − β1(lnCRPdiff) − β2(lnAGPdiff)].

Univariate unadjusted and adjusted (for group) linear regression analyses were used to describe associations between potential “predictor” variables and plasma zinc concentration. The “predictor” variables were decided *a priori* to be of interest, either based on previous associations described in the literature, or because they were considered to potentially have an association with zinc status in toddlers. The variables from baseline that were investigated were: parity, maternal education, socioeconomic status (SES; assessed as the level of household deprivation (NZDep score) [31]), and infant sex. The variables from 12 months that were investigated were: hemoglobin concentration, weight-for-age *z*-score, length-for-age *z*-score, food fussiness score, topical zinc preparation use in the past month; and the intake of: energy, total dietary zinc, phytate, “meat, fish, poultry”, red meat, cow’s milk, dairy (excluding cow’s milk). It was not possible to model breast milk and infant formula intake because there were so many non-consumers, so breast milk, and infant formula (all formulas in New Zealand are iron- and zinc-fortified), were used as dichotomous (i.e., yes vs. no) variables. The age when complementary foods were introduced was also investigated. Although other variables are also known to predict zinc status in childhood (e.g., age, season, time of blood collection, and fasting status), these factors were standardized during data collection and therefore not included in the analyses. Plasma zinc concentration was adjusted for inflammation as described above.

Variables that had an adjusted association of *p* < 0.10 in these univariate analyses, and that were also potentially modifiable, were considered for inclusion in the final multivariate model. This meant that hemoglobin concentration, maternal education, household deprivation, and breast milk consumption were excluded, even though they met the *p* < 0.10 criterion, because they were not considered to be modifiable. Although mothers who were breastfeeding at 12 months could potentially modify the amount their toddler was given, it was not considered to be practically possible to start breastfeeding at 12 months if the toddler had been weaned. Intervention group was not included in the final regression model as it had very little impact on the univariate analyses. Product-moment correlations were used to examine the association between the potentially modifiable “predictors” and plasma zinc concentrations. The final model used robust standard errors to overcome problems with the distribution of some of the variables.

All analyses were conducted using Stata, version 14.2 (StataCorp LP, College Station, TX, USA).

## 3. Results

### 3.1. Participant Characteristics at Baseline

Baseline maternal and infant characteristics are presented in Table 1 for participants who provided a plasma zinc sample (*n* = 115). Participants who provided a plasma zinc sample had similar baseline characteristics to those who did not, with none of the variables in Table 1 differing statistically significantly between those who did and did not provide a sample. Of those who provided a sample, similar proportions were primiparous and multiparous, 73% self-identified as being of New Zealand European ethnicity, and 52% had a university qualification. The level of household deprivation was high for 23% of participants (lower than the 30% expected for the New Zealand population [31]).

### 3.2. Participant Characteristics at 12 Months of Age

Dietary intake, biochemical indices, and anthropometry of toddlers who provided plasma zinc data at 12 months of age are presented in Table 2. Adjusting plasma zinc for acute (CRP) and chronic (AGP) inflammation did not alter the mean plasma zinc concentration at 12 months (Table 2).

**Figure 1:**
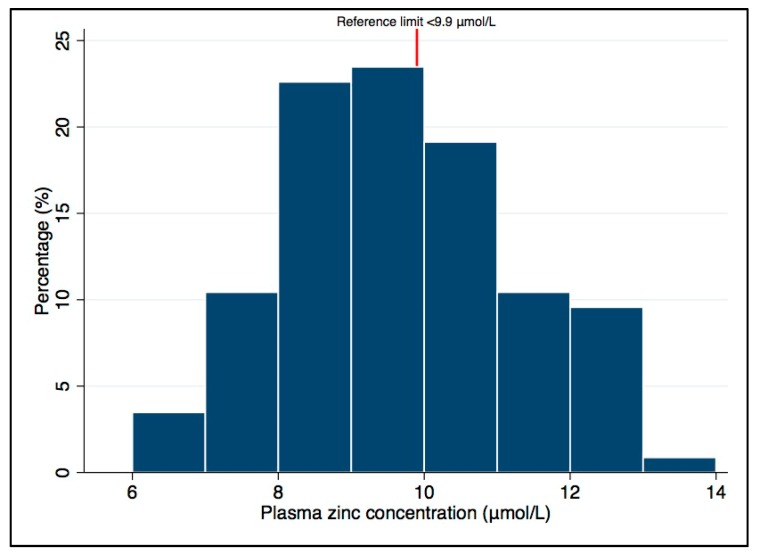
Plasma zinc concentrations (μmol/L) of participants (*n* = 115).

Median (25th, 75th percentiles) intakes of zinc were 4.4 (3.7, 5.4) mg/day and the phytate-to-zinc molar ratio was 5:1 (3.4:1, 7.1:1). The mean (SD) plasma zinc concentration was 9.7 (1.5) μmol/L, with 60% (*n* = 69) of toddlers below the IZiNCG morning non-fasting reference limit of <9.9 μmol/L [37]. The distribution of the plasma zinc values is presented in Figure 1.

The mean (SD) BMI and BMI z-score of participants in the BLISS study groups were 16.9 (1.36) kg/m^2^ and 0.20 (0.89) for the Control group, and 17.3 (1.69) kg/m^2^ and 0.44 (1.13) for the BLISS group [27]. One (0.9%) participant was considered stunted (using the WHO classification of a length-for-age *z*-score < −2 SD [39]) and another (0.9%) was underweight (using the WHO classification of a weight-for-age z-score < −2 SD [39]) at 12 months of age.

### 3.3. Univariate Associations between Potential “Predictor” Variables and Plasma Zinc Concentrations

Unadjusted and adjusted univariate associations with plasma zinc concentration are presented in Table 3. Toddlers of mothers with university education had an almost 1 μmol/L lower plasma zinc concentration than toddlers whose mothers had a school education only, after adjusting for group (*p* = 0.022) (Table 3). Toddlers living in high-deprivation households had an almost 1 μmol/L higher plasma zinc concentration compared with toddlers living in low-deprivation households, after adjusting for group (*p* = 0.043) (Table 3).

### 3.4. Multiple Regression Analysis of “Predictors” of Plasma Zinc Concentrations at 12 Months of Age

The correlation matrix shows the strength of the association between the continuous modifiable “predictor” variables (Table 4). Energy, zinc intake, “meat, fish, poultry”, and red meat were strongly correlated with each other as well as being significantly correlated with plasma zinc, so it was only appropriate for one of them to be used in the final model. Red meat was the variable included in the final model because it was a specific component of the BLISS study intervention [26] and a component of the other variables. Therefore, the final model comprised red meat intake, consumption of infant formula (formulas contained 3–6 mg zinc per 100 g), and food fussiness score (Table 5).

Toddlers had a 0.12 μmol/L higher plasma zinc concentration per 10 g of red meat consumed per day (*p* = 0.004), and toddlers who consumed zinc-fortified infant formula had on average a 0.64 μmol/L higher plasma zinc concentration compared with those who did not consume infant formula (*p* = 0.026) (Table 5). Food fussiness was also significantly associated with plasma zinc concentration. A one-unit increase in food fussiness score (possible score range: 1.0 to 5.0, where highest scores represent increased food fussiness) was associated with a 0.49 μmol/L lower plasma zinc concentration (*p* = 0.028).

The *R*^2^ for the final model was 0.13 (*p* < 0.001) indicating that 13% of the variance in plasma zinc concentration (μmol/L) was explained by these three “predictors”.

## 4. Discussion

In this cross−sectional analysis, a large proportion (60%) of toddlers had plasma zinc concentrations below the recommended reference limit of <9.9 μmol/L [37]. Red meat intake, consumption of infant formula, and food fussiness were significant potentially-modifiable “predictors” of zinc status at 12 months of age.

Whether the high proportion of toddlers with plasma zinc values below the reference limit is of concern is uncertain. The current reference limit applied here was not based on data for children less than three years of age [2] and, hence, may be inappropriate for toddlers aged 12 months [40,41].

Several variables were significantly associated with plasma zinc concentration in the univariate analysis including hemoglobin, maternal education, household deprivation, dietary intakes of: energy, zinc, “meat, fish, poultry”, red meat, and consumption of breast milk and infant formula. In contrast to findings from previous studies [18,24,42,43], no association was seen between plasma zinc concentration and growth indicators (length-for-age and weight-for-age *z*-scores), or dietary phytate intake. However, in the final regression model, the variables that were significantly associated with plasma zinc concentration at 12 months, and were potentially modifiable, were red meat intake, consumption of zinc-fortified infant formula, and food fussiness score.

The magnitude of the association between plasma zinc concentration and red meat was small (0.12 μmol/L per 10 g of intake). This small effect can be illustrated by the increase in red meat consumption required to theoretically produce an increase in plasma zinc concentration from the observed mean of 9.7 μmol/L to the recommended reference level of 9.9 μmol/L [37]. At 17 g/day, this increase in red meat intake is substantially higher than the median intake of this group (4.4 g/day), indicating that such an increase would be challenging. Although a previous intervention in New Zealand toddlers reported a 17 g per day increase in red meat consumption, the meals were provided ready-prepared and free of charge to the parents [44]. This suggests that modifying red meat intake alone is unlikely to be sufficient to increase plasma zinc concentrations meaningfully at 12 months of age. Although there have been concerns about the possible health effects of excessive intakes of red meat in adults [45], we are aware of no published studies that have investigated whether there may be detrimental effects of an increased intake of red meat in infants and toddlers. An increase in red meat intake of 17 g/day, as discussed in the current study, would result in total intakes of approximately 150 g/week—substantially lower than the recommended safe level for adults of 500 g/week (no corresponding figures exist for infants). The saturated fat content of red meat may also be a concern. However, in adults, an increased intake of lean red meat in an otherwise healthy diet does not appear to adversely affect serum lipids [46].

Toddlers consuming zinc-fortified infant formula had a 0.64 μmol/L higher plasma zinc concentration than toddlers who did not consume formula. While consuming infant formula may seem achievable for toddlers, it is important to consider whether the consumption of infant formula would then replace other milk feeding (particularly breast milk), and the cost. It is also not clear from the analyses we have been able to carry out here how much infant formula is needed to have a meaningful impact on zinc status—presumably there is a dose−response relationship of some sort, and intakes were as high as 908 mL per day in some of these toddlers.

**Figure 2:**
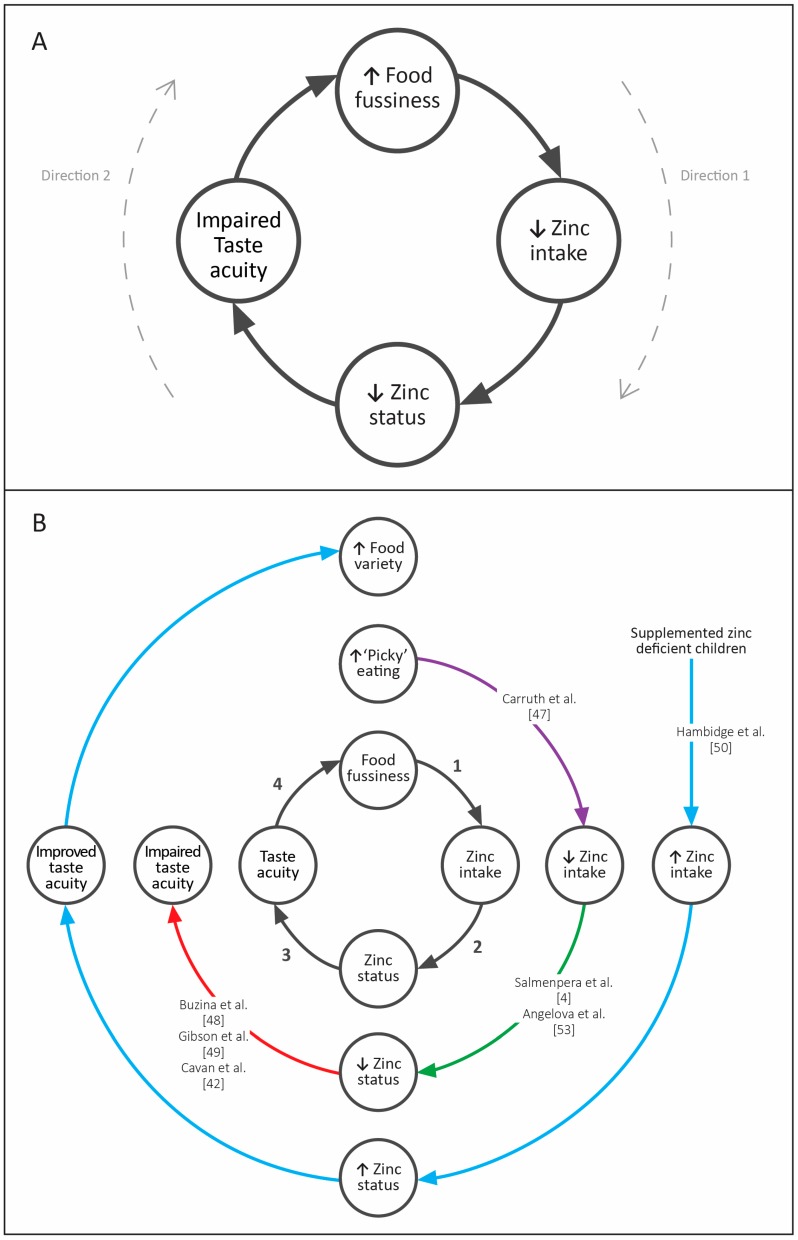
Four plausible pathways in the association between food fussiness and zinc status (**A**); with existing evidence for these associations [4,42,47,48,49,50,53] (**B**)

The relationship between food fussiness and zinc concentrations is potentially exciting. A one-point lower food fussiness score was associated with a plasma zinc concentration that was 0.49 μmol/L higher in this group of toddlers. This suggests that either a decrease in food fussiness score could result in an increase in plasma zinc concentration (Figure 2A: direction 1), or an increase in plasma zinc concentration could result in a lower food fussiness score (Figure 2A: direction 2). Unfortunately, it is not possible to determine from this observational analysis whether food fussiness caused lower plasma zinc concentration, or vice versa. There is very limited research in this area, but it does appear that increased food fussiness may result in decreased zinc intake (Figure 2B: pathway 1) [47] and there is a plausible mechanism for this association—if an infant or toddler is more food fussy, then they may eat fewer foods that are high in zinc, and may also consume fewer foods that enhance zinc absorption (e.g., meat). In turn, decreased zinc intakes may result in poorer zinc status (Figure 2B: pathway 2). However, there is also evidence for the opposite pathway from low zinc status to increased food fussiness. Lower zinc status may result in impaired taste acuity in children [42,48,49] which may, in turn, result in higher food fussiness [50] (Figure 2B: pathways 3 and 4).

The only way to resolve the uncertainty about the direction of the association reported in the current study would be to conduct a randomized controlled trial of zinc supplementation, with the measurement of changes in taste acuity and food fussiness; or an intervention to reduce food fussiness (such as the behavioral intervention by Birch et al. [51], which achieved an improvement in children’s food preference by increasing the frequency of their exposure to a food) with measurement of the subsequent impact on zinc intake and plasma zinc concentration.

The final regression model comprising red meat intake, infant formula consumption, and food fussiness, explained 13% of the variance in plasma zinc concentrations. This highlights that many other factors are contributing to zinc status in toddlers that were not included in this final model. More work is necessary to determine further factors that have the potential to affect plasma zinc concentrations in this age group, and whether any of these are potentially modifiable.

This study has a number of strengths, including rigorous dietary data collection using weighed diet records, a method that is recommended for estimating dietary intakes of very young children [52]. The quality of the dietary assessment data is reflected in our ability to detect a significant association between dietary zinc intake and zinc status. We used strict trace element-free methods to collect and separate blood samples for determining plasma zinc concentrations, as recommended by IZiNCG [40]. Additionally, we were able to minimize variability due to fasting status by using a rigorous pre-sample protocol that included encouraging parents to feed their child milk 90 min before the blood test, and then no other food or drink until after the blood sample was collected; and to minimize the impact of infection on plasma zinc concentrations, by delaying the blood test for 14 days if the child was unwell.

It is important to note the limitations of the current study which include that this was a cross-sectional secondary analysis using data from the BLISS study—a randomized, controlled trial that was not specifically designed to determine predictors of zinc status. Additionally, although the biochemical and dietary data were both collected at, or soon after, the toddlers turned 12 months of age, the dietary data were collected over a three-week period, and some participants’ blood samples were delayed if they had been unwell, so the intakes presented here may not be a true reflection of dietary intakes immediately before the blood sample was collected. Lastly, conclusions from this study should be treated with caution as this was an observational study, so causation and the direction of associations cannot be determined.

## 5. Conclusions

In this cross-sectional analysis, intake of red meat and the consumption of zinc-fortified infant formula were positively associated with plasma zinc concentrations, whereas food fussiness score was inversely associated with plasma zinc. Although higher intakes of red meat and the consumption of infant formula are potentially achievable in the diets of toddlers, it is important to consider the potential barriers associated with increasing intakes of both of these foods—particularly the possible impact on breastfeeding, cost, and parents’ and toddlers’ willingness to modify their behavior. This analysis provides compelling evidence for an association between food fussiness and zinc status, however, further studies are required to determine the direction of this association.

## Figures and Tables

**Table 1:** Maternal and infant baseline characteristics of participants who provided plasma zinc data at 12 months of age ^1^.

	Total (*n* = 115)
**Maternal and Household Variables at Baseline**	
Maternal parity	
First child	44 (38)
Two children	47 (41)
Three or more children	24 (21)
Maternal ethnicity	
New Zealand European	84 (73)
Māori	22 (19)
Other ^2^	9 (8)
Maternal education	
School only	33 (29)
Post-secondary	22 (19)
University	60 (52)
Household deprivation ^3^	
1–3 (Low)	30 (26)
4–7	59 (51)
8–10 (High)	26 (23)
**Infant variables at baseline**	
Sex	
Female	61 (53)
Male	54 (47)
Group ^4^	
Control	58 (50)
BLISS	57 (50)

^1^ Data presented as *n* (%). ^2^ Other ethnicities were Asian and Pacific. ^3^ Household deprivation categorized using the NZDep scale in which decile 1 indicates the lowest level of deprivation and 10 indicates the highest [31]. ^4^ As part of the BLISS randomized controlled trial, participants were randomized to either the Control or BLISS group after stratification for maternal education and parity [27].

**Table 2:** Characteristics of toddlers who provided plasma zinc data at 12 months of age (*n* = 115).

	Median (25th, 75th) ^1^
**Biochemical Variables**	
Unadjusted plasma zinc, μmol/L (mean (SD))	9.7 (1.5)
Adjusted plasma zinc, μmol/L (mean (SD)) ^2^	9.7 (1.5)
Hemoglobin, g/L (mean (SD)) ^3^	117 (8.7)
C-reactive protein, mg/L	0.1 (0.0, 0.5)
α_1_-acid glycoprotein, g/L	0.61 (0.47, 0.87)
**Dietary variables ^4^**	
Energy, kJ/day	3543 (3090, 4168)
Zinc, mg/day	4.4 (3.7, 5.4)
Phytate, mg/day	230 (150, 318)
Phytate:zinc molar ratio ^5^	5.0 (3.4, 7.1)
“Meat, fish, poultry”, g/day	18.9 (9.5, 30.5)
Red meat, g/day	4.4 (0, 11.5)
Cow’s milk, g/day	21.6 (5.9, 132)
Dairy, g/day ^6^	39.9 (9.5, 73.9)
Breast milk (*n* (%))	
No	47 (45)
Yes	57 (55)
Infant formula (*n* (%))	
No	59 (57)
Yes	45 (43)
**Other variables**	
Weight, kg (mean (SD)) ^7^	9.8 (1.1)
Length, cm (mean (SD)) ^7^	75.8 (2.6)
Weight-for-age z-score (mean (SD)) ^7,8^	0.37 (0.96)
Length-for-age z-score (mean (SD)) ^7,9^	0.26 (0.93)
Food fussiness score (mean (SD)) ^7,10^	2.1 (0.6)
Age complementary foods introduced, weeks (mean (SD))	23.5 (3.6)
Topical zinc preparation use in the past month (*n* (%))	
No	56 (49)
Yes	59 (51)

^1^ Data presented as median (25th, 75th), unless otherwise specified. ^2^ Adjusted plasma zinc = exp[unadjusted lnplasmazinc − (regression coefficient for lnCRP) * (lnCRPdiff) − (regression coefficient for lnAGP) * (lnAGPdiff)] from Larson et al. [38]. ^3,4^ Available data for ^3^ *n* = 114, ^4^ *n* = 104. ^5^ Calculated as [phytate (mg)/660]/[zinc (mg)/65.4]. ^6^ Excludes cow’s milk. ^7^ Available data for *n* = 114. ^8^ Weight-for-age *z*-score calculated using the World Health Organization child growth standards reference data [34]. ^9^ Length-for-age *z*-score calculated using the World Health Organization child growth standards reference data [34]. ^10^ Food fussiness was determined using the six questions on food fussiness from the Children’s Eating Behaviour Questionnaire [32]. Lowest score: 1.0, highest score: 5.0.

**Table 3:** Univariate associations between potential “predictor” variables and plasma zinc concentration (μmol/L) at 12 months of age.

		Change in Plasma Zinc Concentration (μmol/L) ^1^ for Each Unit Change in the Potential “Predictor”			
		Unadjusted		Adjusted for Group	
	*n* (%)	B (95% CI)	*p*	B (95% CI)	*p*
**Biochemical variables**					
Hemoglobin, g/L	114 (99)	0.05 (0.01, 0.08)	**0.005**	0.05 (0.02, 0.08)	**0.004**
**Dietary variables**					
Energy, kJ/day	104 (90)	0.00 (−0.00, 0.00)	**0.07**	0.00 (−0.00, 0.00)	**0.07**
Zinc, mg/day	104 (90)	0.23 (0.07, 0.39)	**0.005**	0.23 (0.07, 0.39)	**0.005**
Phytate, mg/day	104 (90)	0.00 (−0.00, 0.00)	0.70	0.00 (−0.00, 0.00)	0.71
“Meat, fish, poultry”, g/day	104 (90)	0.02 (0.00, 0.03)	**0.015**	0.02 (0.00, 0.03)	**0.016**
Red meat, g/day	104 (90)	0.02 (0.00, 0.03)	**0.015**	0.02 (0.00, 0.03)	**0.016**
Cow’s milk, g/day	104 (90)	0.00 (−0.00, 0.00)	0.66	0.00 (−0.00, 0.00)	0.69
Dairy, g/day ^2^	104 (90)	−0.00 (−0.01, 0.00)	0.50	−0.00 (−0.01, 0.00)	0.54
Breast milk					
No	47 (45)	1.00 (reference)	-	1.00 (reference)	-
Yes	57 (55)	−0.53 (−1.11, 0.06)	**0.077**	−0.52 (−1.11, 0.06)	**0.080**
Infant formula					
No	59 (57)	1.00 (reference)	-	1.00 (reference)	-
Yes	45 (43)	0.76 (0.18, 1.33)	**0.010**	0.77 (0.19, 1.34)	**0.010**
**Other variables**					
Maternal parity					
First child	44 (38)	1.00 (reference)	-	1.00 (reference)	-
Two children	47 (41)	−0.17 (−0.81, 0.47)	0.60	−0.16 (−0.80, 0.48)	0.62
Three or more children	24 (21)	−0.41 (−1.19, 0.36)	0.29	−0.39 (−1.18, 0.39)	0.32
Maternal education					
School only	33 (29)	1.00 (reference)	-	1.00 (reference)	-
Post-secondary	22 (19)	−0.33 (−1.15, 0.49)	0.43	−0.35 (−1.18, 0.48)	0.41
University	60 (52)	−0.77 (−1.42, −0.12)	**0.020**	−0.77 (−1.42, −0.11)	**0.022**
Household deprivation ^3^					
1–3 (Low)	30 (26)	1.00 (reference)	-	1.00 (reference)	-
4–7	59 (51)	0.09 (−0.60, 0.78)	0.80	0.07 (−0.61, 0.76)	0.83
8–10 (High)	26 (23)	0.80 (0.02, 1.56)	**0.044**	0.80 (0.03, 1.57)	**0.043**
Sex					
Male	54 (47)	1.00 (reference)	-	1.00 (reference)	-
Female	61 (53)	−0.21 (−0.78, 0.36)	0.47	−0.23 (−0.81, 0.34)	0.42
Weight-for-age *z*-score ^4^	114 (99)	0.02 (−0.28, 0.32)	0.91	0.02 (−0.28, 0.32)	0.90
Length-for-age *z*-score ^5^	114 (99)	0.14 (−0.16, 0.45)	0.35	0.16 (−0.15, 0.47)	0.32
Food fussiness score ^6^	114 (99)	−0.44 (−0.89, 0.02)	**0.06**	−0.41 (−0.88, 0.06)	**0.09**
Age complementary foods introduced, weeks	115 (100)	0.03 (−0.05, 0.11)	0.39	0.03 (−0.05, 0.11)	0.47
Topical zinc preparation use in the past month					
No	59 (51)	1.00 (reference)	-	1.00 (reference)	-
Yes	56 (49)	−0.07 (−0.64, 0.50)	0.82	−0.07 (−0.64, 0.50)	0.81

Bold indicates *p* < 0.10. ^1^ Adjusted plasma zinc = exp[unadjusted lnplasmazinc − (regression coefficient for lnCRP) * (lnCRPdiff) − (regression coefficient for lnAGP) * (lnAGPdiff)] from Larson et al. [38]. ^2^ Excludes cow’s milk. ^3^ Household deprivation categorized using the NZDep scale in which decile 1 indicates the lowest level of deprivation and 10 indicates the highest [31]. ^4^ Weight-for-age *z*-score calculated based on the World Health Organization standards [34]. ^5^ Length-for-age *z*-score calculated based on the World Health Organization standards [34]. ^6^ Food fussiness was determined using the six questions on food fussiness from the Children’s Eating Behaviour Questionnaire [32]. Lowest score: 1.0, highest score: 5.0.

**Table 4:** Correlations (r) between potentially modifiable “predictor” variables, and between these continuous variables and plasma zinc concentration.

			Potentially Modifiable “Predictor” Variables					
		Plasma Zinc	Energy	Zinc	Red Meat	MFP	Infant Formula	Food Fussiness
**Potentially modifiable “predictor” variables**	**Plasma zinc**	-						
	**Energy**	0.18	-					
	**Zinc**	0.27 *	0.82 **	-				
	**Red meat**	0.24 *	0.59 **	0.73 **	-			
	**MFP**	0.24 *	0.53 **	0.70 **	0.70 **	-		
	**Food fussiness**	−0.18	−0.15	−0.07	−0.01	0.01	0.10	-

Abbreviations: MFP, “meat, fish, poultry”. * *p* < 0.05, ** *p* < 0.001.

**Table 5:** Multiple regression analysis of “predictors” of plasma zinc concentrations at 12 months of age (*n* = 103).

	Change in Plasma Zinc Concentration (μmol/L) ^1^ for Each Unit Change in the “Predictor”	
	B (SE)	*p*
Red meat intake, 10 g/day	0.12 (0.04)	**0.004**
Infant formula		
No	1.00 (reference)	-
Yes	0.64 (0.28)	**0.026**
Food fussiness score ^2^	−0.49 (0.22)	**0.028**

Bold indicates a statistically significant difference at *p* < 0.05. ^1^ Adjusted plasma zinc = exp[unadjusted lnplasmazinc − (regression coefficient for lnCRP) * (lnCRPdiff) − (regression coefficient for lnAGP) * (lnAGPdiff)] from Larson et al. [38]. ^2^ Food fussiness was determined using the six questions on food fussiness from the Children’s Eating Behaviour Questionnaire [32]. Lowest score: 1.0, highest score: 5.0.
